# Temperance and Light Wines

**Published:** 1907-08-24

**Authors:** 


### The Hospital

A Journal of

The Medical Sciences and Hospital Administration.

New Series. No. 22, Vol. I. [No. 1094, Vol. XLII.] SATURDAY, AUGUST 24, 1907.

### TEMPERANCE AND LIGHT WINES.

Our attention has been drawn to two leading articles in the *Temperance Record* criticising our special report on the chemical composition and dietetic value of light wines. We have the greatest respect for and sympathy with the objects of the *Temperance Record*, and the numerous quotations which our contemporary makes from our report are in themselves evidence that we are quite alive to the harm done by the abuse of alcohol. At the same time, since our article was meant primarily for the profession, we did not think it was incumbent upon us to devote space to the admittedly important question, whether from the point of view of the race, the good effects of alcohol were not over-balanced by its harmful ones. Further, it was not our function to inquire whether or not nothing but evil accrues from the use of alcohol, nor whether its prescription should be entirely abandoned by the medical profession. Indeed, our object was primarily not alcohol at all, but wine, and essentially to see how different wines affected the different digestive processes, and from this to infer their doses and some of their uses and abuses. We thought, when initiating this report, that one must be scientific before one is ethical, and that at any rate, dealing as we do with the medical profession, our first function was to endeavour to find out to the best of our power the truth, and, having found it, to tell it as simply as possibly without any pro-alcoholic or anti-alcoholic garnishing.

Having dealt briefly with what was roughly our object, we will deal *seriatim* with the criticisms. The first serious criticism is one based upon our remark that one can only get sufficient alcohol from wine to produce its full action of making one drunk by taking sufficient extract to make one ill. If the Editor had read the context he would have found that this was, so to speak, the culminating remark of a comparison between the action of spirits and wine, the former being an almost pure alcoholic beverage and giving the toxic effect of pure alcoholism, the latter containing a relatively large amount of extract. The extractives of wine do disagree with many patients, and this is well known, and is the reason why whisky and other spirits are so frequently resorted to. Our point, roughly, was this, that from the point of view of the action of the alcohol, wine contained a protective mechanism in the shape of by-products, and if dipsomanias took wine as their intoxicating drink, they would not take so much alcohol as if they took spirits, because the extract in wine would make them ill before they got what to them—namely, the drunkard—was the full effect of the alcohol.

The next criticism concerns the eternal question as to whether alcohol is or is not a food. As a matter of fact, we do not regard this question of much importance. We never say, as we are represented to do, that a food " merely produces energy by simple oxidation." We do not know what " simple " oxidation is: perhaps the Editor will tell us. Matters scientific are not always quite so simple as they appear. The Editor informs us that in order for a food to be a food, it must build up the tissues; if this means anything scientifically, it means it must contain nitrogen; therefore neither sugar nor fat are foods. Alcohol, in suitable doses, and sugar, are forms of energy, and bear the same relation to beef steak as a crossed cheque does to a sovereign; the body, like the spender, can get at the real value of the latter easier than at that of the former. We quite agree with our contemporary that wine is taken in the main because it is pleasurable, and that only too often not sufficient thought is given to what this pleasure may cost. That is precisely why we undertook our inquiry, and endeavoured, as far as was possible under the conditions set, not only to indicate the differences between different kinds of wine, but also approximately their doses.

The next remark of our contemporary is again, we are afraid, due to a regrettable neglect to read the article criticised. We are upbraided for calling a certain Australian wine containing 16.19 per cent. of alcohol a light wine; in two places in the article we expressly say that this wine, and, indeed, this class of wine, are in no sense comparable to French clarets, and cannot be called light wines. We are taking all the criticisms of our contemporary most seriously, as we should be exceedingly sorry if any inference of ours should in any way militate against the cause of temperance, which we have every desire to assist.

The *Temperance Record* criticises severely our statement that light wines are essentially temperance beverages. We admit at once that that statement was relative, and loses some of its significance when lifted bodily out of its context. When we made the statement we were comparing wines with spirits, and we obviously referred to wines as recommended to be used by the report. We are, however, prepared to defend the statement upon absolute grounds. We assume that the editor admits, with the excise authorities, that a beverage, for instance, like ginger beer, which may and often does contain 2 per cent. of alcohol, is a non-alcoholic beverage. The report recommends the use of about 20-25cc. of alcohol contained in 200 or 250cc. of light wine at meal times, diluted with an equal quantity of water; in other words, it recommends the use of a 5 per cent. solution of alcohol, or a beverage containing rather more than twice the alcohol contained in ginger beer. We do not think that the term "essentially a temperance beverage" is very inappropriate to such a drink. Leaving the question of tannin, which is far more important in regard to tea-drinking than to wine-drinking, we come to the last essential criticism, namely, the charge made against us that the experiments were only made in test tubes, and that we made no controls. We quite admit that the experiments were made in test-tubes, and this to the lay mind is a very ear-catching criticism. Since, however, practically all our knowledge of the chemistry of digestion, which has subsequently been confirmed by clinical observation, is derived from experiments made in test-tubes, or, at any rate, glass vessels, we make no apology for the technique. The results of these experiments are as clear and traducible as any other class of chemico-physiological experiment. With regard to the question of controls, we must again charge our contemporary with careless reading. We have made and published controls made with simple alcohol and also water. The control with the $CO_2$ and by-products of champagne we did not make because it would be quite impossible to do so; further, there was no necessity for making it. If we know by control, which we do, that the alcohol in the strength in which it would be present in the champagne digestive mixture would not produce the effect the champagne produced, it is reasonable to infer that the stimulated digestion of proteids produced by the champagne was, at any rate, partly due, which is all we say, to the non-alcoholic constituents of the wine.

As we have said above, we have considered the article in the *Temperance Record* carefully because we believe in, and wish to aid, by ascertaining and emphasizing truth, the cause of temperance, but we fear that irresponsible criticism, based upon inaccurate perusal of the work criticised, will bring any cause, however praiseworthy, into disrepute.

